# Spatial distribution pattern of colonized native semi-shrubs in two artificial vegetation restoration patterns in Mu Us sandy land, North China

**DOI:** 10.1371/journal.pone.0304204

**Published:** 2024-06-06

**Authors:** Ercha Hu, Runhong Gao

**Affiliations:** 1 College of Forestry, Inner Mongolia Agricultural University, Hohhot, Inner Mongolia, China; 2 Inner Mongolia Academy of Forestry Sciences, Hohhot, Inner Mongolia, China; Ningbo University, CHINA

## Abstract

Vegetation construction is a key process for restoring and rehabilitating degraded ecosystems. However, the spatial pattern and process of native plants colonized by different vegetation restoration methods in semi-arid sandy land are poorly understood. In this study, two artificial vegetation restoration patterns (P1: row belt restoration pattern of *Salix matsudana* with low coverage; P2: a living sand barrier pattern of *Caryopteris mongolica* with low coverage) were selected to analyze the spatial distribution pattern and interspecific association of the colonizing native shrubs. The effects of the two restoration models on the spatial patterns of the main native semi-shrubs of the colonies (i.e., *Artemisia ordosica* and *Corethrodendron lignosum* var. *leave*) were studied using single variable and bivariate transformation point pattern analysis based on Ripley’s *L* function. Our results showed that two restoration patterns significantly facilitated the establishment of *A*. *ordosica* and *C*. *lignosum* var. *leave*, with their coverage reaching 17.04% and 22.62%, respectively. In P1, the spatial distribution pattern of colonial shrubs tended to be a random distribution, and there was no spatial correlation between the species. In P2, the colonial shrub aggregation distribution was more dominant, and with the increase in scale, the aggregation distribution changed to a random distribution, whereas the interspecific association was negatively correlated. The differences in the spatial distribution patterns of colonized native semi-shrubs in these two restoration patterns could be related to the life form of planted plants, configuration methods, biological characteristics of colonized plants, and intra- and interspecific relationships of plants. Our results demonstrated that the nurse effect of artificially planted vegetation in the early stage of sand ecological restoration effectively facilitated the near-natural succession of communities. These findings have important implications for ecological restoration of degraded sandy land in the semi-arid region of northern China.

## Introduction

Desertification is a major environmental problem in arid, semi-arid, and dry sub-humid areas, and has a widespread impact on regional society and economy [1, 2]. China is one of the most seriously desertified countries in the world, and desertification has affected more than 25% of the national land area [3]. The Mu Us sandy land is located in the agro-pastoral ecotone of semi-arid regions of northern China, where ecosystems are typically fragile and sensitive to periodic drought [4] and unsustainable human activities [5]. For decades, this area has suffered from serious desertification, primarily due to improper exploitation and wind erosion [5, 6] implying that the restoration of the degraded ecosystem is urgently needed, as well as this being a big challenge [7].

Vegetation construction is commonly used as a windbreak to stabilize sand [8], and is proven to be one of the most low-cost and effective ecological restoration measures [9, 10]. It is generally believed that vegetation construction plays an important role in reducing wind erosion, improving soil nutrient content, and regulating climate [11]. However, inappropriately implemented vegetation restoration may cause negative ecological effects, while large scale and high-density artificial afforestation may lead to the reduction of deep percolation and groundwater recharge in arid and semi-arid areas [12–14]. Plant interactions and their underlying mechanisms must be understood when formulating a vegetation restoration plan. Further research is needed to clarify the processes and mechanisms of plant community establishment under different restoration patterns, to guide the selection of appropriate technologies.

Intra- and inter-species interactions are key processes that affect the structure and dynamics of plant communities [15, 16]. Plant interactions include positive and negative effects, which together with environmental factors affect the spatial patterns and succession of communities [17, 18]. The “facilitation” model suggests that established vegetation can facilitate the integration of new individuals into the community, while the “inhibition” model suggests that once the earlier colonists are established, they will continue to exclude or restrain later colonists until the former is dead or damaged [19]. Many studies have demonstrated that facilitation and inhibition are common in natural communities; they determine the direction and speed of community succession [20, 21]; their manipulation can be used to affect the development of degraded ecosystems into desired communities [22]. The life form of interacting species largely influenced the interaction outcomes [23]. Shrubs had large facilitative effects, while herbs had strong inhibitive effects, especially on other herb species [23]. The nurse plant effect of woody plants on their underlying herbs have been well documented in desert plant communities with different life forms [24, 25]. In most cases, competition plays a dominant role in a productive environment with more available resources, while facilitation effects would be more important under greater environmental stresses [26–28]. Many empirical studies and meta-analyses have been conducted to test this prediction in arid and semiarid ecosystems [23, 29]. However, the role of positive and negative interactions in different artificial vegetation restoration measures remains uncertain. An examination of spatial distribution patterns and interactions among species in different artificial vegetation restoration measures would provide valuable information on how communities are shaped, which is important for the restoration and management of degraded ecosystems.

A spatial distribution pattern is the specific manifestation of a plant community structure, mainly determined by competition (negative effect) and facilitation (positive effect) [30]. If facilitation is the main factor determining the spatial pattern of plants, distribution tends to be aggregated, and if competition is more dominant, the distribution tends to be uniform. If both facilitation and competition are absent, the distribution tends to be random [31]. As a typical example, the seedlings of trees or shrubs are commonly found clustered near the adult individuals [32]. Studying the spatial distribution pattern of species is helpful in understanding the specific performance of plant interactions in life strategies such as recruitment, growth, death, and resource utilization [33, 34].

The reconstruction of artificial vegetation is an effective measure for the restoration of degraded ecosystems in sandy land. However, artificial promotion of near-natural vegetation restoration has not received enough attention. To better understand and clarify the effects of different artificial vegetation restoration patterns on native plant restoration in Mu Us sandy land, two artificial vegetation restoration patterns were selected to analyze the spatial distribution pattern and interspecific association of the colonized native shrubs. The objectives of this study were: (1) to analyze the spatial distribution patterns and inter-species associations of naturally restored native shrubs *A*. *ordosica* and *C*. *lignosum* var. *leave* in two vegetation restoration patterns, and (2) to reveal the recruitment mechanisms of native shrubs in the early stage of artificial vegetation restoration in sandy land. The research can provide a scientific base and theoretical support model for vegetation restoration in Mu Us sandy land.

## Materials and methods

### Study sites and species

**Fig 1:**
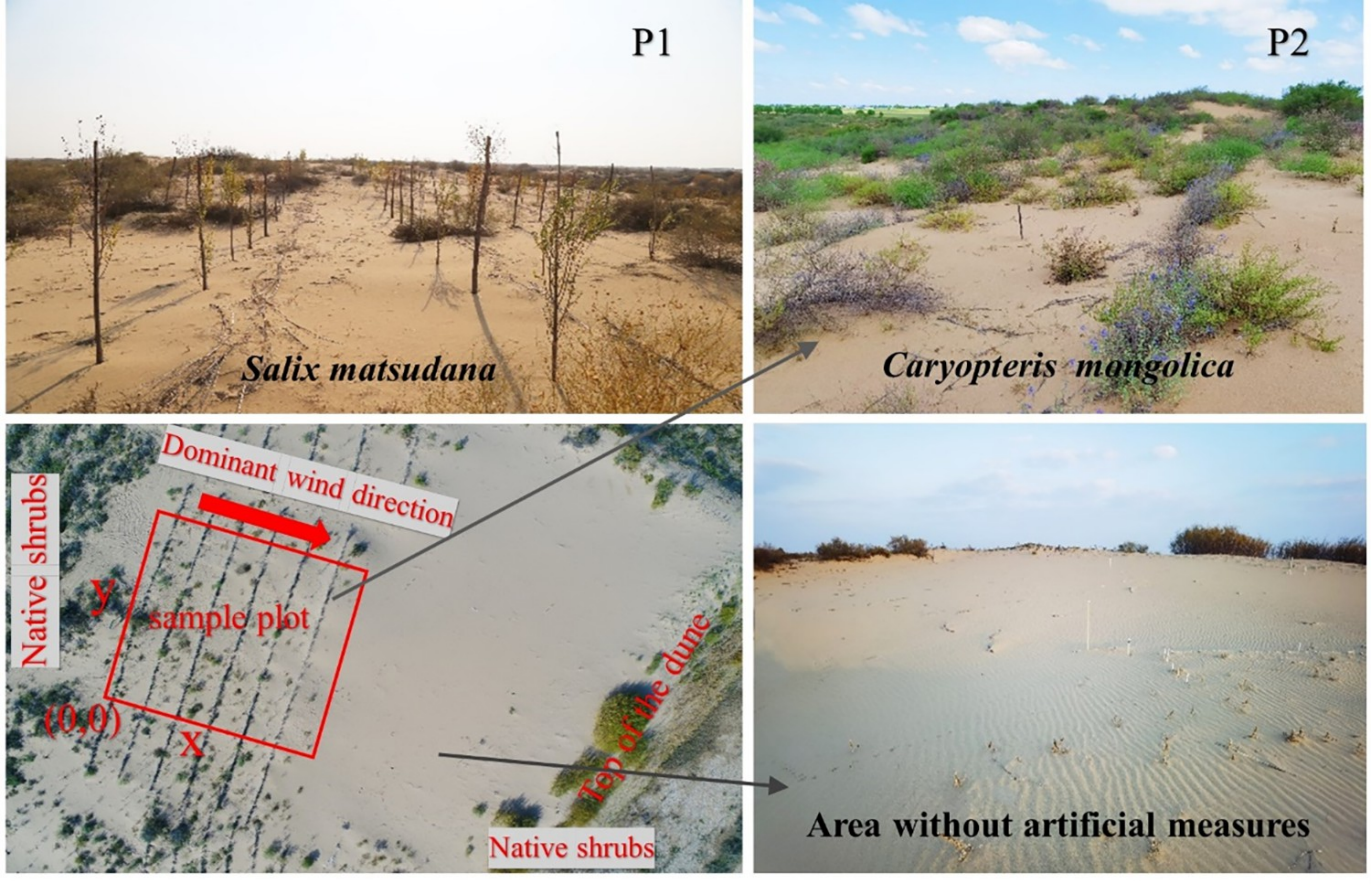
The current landscape of the sampling sites.

The study area was located in the Wushen Banner (109°15’37.71″E, 38°51’5.07″N), an agro-pastoral region of Inner Mongolia, northern China (Fig 1). The area lies in the belly of the Mu Us sandy land and is characterized by a mid-temperate, semi-arid continental monsoon climate. The mean annual temperature is approximately 6.8°C, and the annual precipitation is 350–400 mm, 62% of which falls from July to September. The average wind speed is 2.7–3.5 m/s, with predominantly northwesterly winds in the spring. The terrain is relatively flat, with fixed, semi fixed, semi mobile dunes and inter dune lowlands alternately distributed. The dominant vegetation comprises *A*. *ordosica*, *C*. *lignosum* var. *leave*, *Salix psammophila*, and other desert shrubs.

Since the 1970s, an aerial seeding sand control project has been implemented in Mu Us sandy land. After more than 40 years of restoration and succession, fixed sand dunes and semi fixed sand dunes with *A*. *ordosica*, *C*. *lignosum* var. *leave* and *S*. *cheilophila* as the dominant species were created but due to human factors, such as overgrazing and the effects of climatic drought, resulting in degradation of some areas [4]. Two ecological restoration patterns, *S*. *matsudana* plantations and *C*. *mongolica* living sand barriers, were selected to study the spatial patterns of colonial native shrubs. Both sites were located in the middle and lower parts of the connected dunes, with similar environmental conditions, and each pattern area is approximately 0.5 hm^2^. Project implementation began in 2017. Before restoration, the two plots were mobile dunes without vegetation but from the third year it was apparent that the bare sand was gradually being colonized by the native dominant shrubs *A*. *ordosica* and *C*. *lignosum* var. *leave*. After 4 years of restoration, the number and crowns of the native shrubs *A*. *ordosica* and *C*. *lignosum* var. *leave* had increased significantly, but herbs were still rare, with only a small number of annual herbs, such as *Agriophyllum squarrosum* and *Corispermum hyssopifolium*, being found. Because of this, these herbs were not used in this study.

*A*. *ordosica* is a semi-shrub, 50–100 cm in height, with a strong woody stock, much branched. The stems of *A*.*ordosica* seedlings are brown or dark purple, while the adults are dark gray or dark brown. *A*. *ordosica* has tap root system, and its root system is mainly distributed in the upper 30 cm of the sand, while its main roots may reach 1–3 m deep. The recruitment of *A*. *ordosica* is generally realized by seed reproduction and is characterized by R-strategy species, and the plant begins to reproduce at the age of 2–3 years. *A*. *ordosica* has a high flowering and seed setting rate, and the volume and quality of achenes are very small. These are also the special structures of *A*. *ordosica* seeds to adapt to wind-borne transmission. Small seeds can also reduce the need for water for germination, thereby increasing the germination rate of seeds under drought conditions [35, 36].

*C*. *lignosum* var. *leave*, semi-shrub, 40–80 cm tall, much branched, seedling stems green, while adults gray white or gray brown. *C*. *lignosum* var. *leave* is a perennial deciduous semi-shrub with strong adaptability and can grow on extremely arid and barren semi-fixed and fixed sandy land. *C*. *lignosum* var. *leave* has strong natural regeneration ability, recruitment includes seed reproduction and clonal reproduction, and clonal reproduction plays an important role in its life history. *C*. *lignosum* var. *leave* has underground rhizomes, and buds are on the nodes and tops of rhizomes. These buds can grow new plants upward. In natural habitats, genet generally grows for 2 to 3 years and then sprouts 1 to several rhizomes at the root neck. These rhizomes grow horizontally underground for a period of time and then turn upward to develop into daughter ramets, which continuously produce new plants. The study of clonal growth of *C*. *lignosum* var. *leave* in Mu Us Sandy Land found that the average number of underground rhizomes per individual plant of *C*. *lignosum* var. *leave* can reach 14, the total length is 14.36 m, and the average length of single underground rhizome is about 0.82 m [37, 38].

### Experimental design and field surveys

A set of 30 m × 30 m sampling plots was established in each of the two restoration patterns, using a coordinate system with due east as the x-axis (perpendicular to rows), and due north as the y-axis (parallel to rows), with the southwest vertex of the plot as the origin (0,0) (Fig 1). Each sample plot was divided into 9 contiguous 10 m × 10 m subplots. In each subplot, the relative position coordinates, canopy length (*L*), canopy width (*W*), and canopy height (*H*), ground diameter (or diameter at breast height) and growth status of each *A*. *ordosica* and *C*. *lignosum* var. *leave* was measured. The canopy was assumed to be an ellipse, and its area calculated using the formula (π × *L* × *W*)/4 (Table 1). Previous research has found a significant relationship between the canopy width, height of shrubs, and the development stage of shrubs in arid environments [39, 40]. Using growth indexes such as height, canopy, branch number and lignification degree of *A*. *ordosica* and *C*. *lignosum* var. *leave*, combined with the results from previous studies[10, 35], individual plants were divided into two groups, namely adults and seedlings (seedling group: average heights and crown were less than 30 cm, single plant, low lignification, and the stems of *A*. *ordosica* were brown or dark purple, and the stems of *C*. *lignosum* var. *leave* were green; adult group: average heights and crown were more than 30 cm, and the stems of *A*. *ordosica* were dark gray or dark brown, and the stems of *C*. *lignosum* var. *leave* were gray white or gray brown).

**Table 1:** General situation of sampling sites.

Patterns	species	Configuration mode (Plant row spacing) /m	Habitats	Height/cm	Diameter at breast height (Ground diameter) /mm	Crown/m^2^	Coverage/%		
							Artificial plants	Colonized shrubs	Herbs
P1	*S*. *matsudana*	2×4	Windward slope of sand dunes	263.14±32.11	35.51±10.88	0.39±0.15	2.67	17.04	<1
P2	*C*. *mongolica*	0.5×5	Windward slope of sand dunes	57.50±16.46	9.04±5.16	0.29±0.13	9.60	22.62	<1

During afforestation, 6 wind erosion piles were evenly set between the rows of the two sample plots, and 6 wind erosion piles were set up in the downwind bare sand dunes as a control. The initial height of the wind erosion pile was 40 cm. The height of the wind erosion pile was measured during the vegetation survey to obtain the amount of wind erosion.

### Data analysis

Spatial distribution patterns were analyzed using Ripley’s *L*-function [41, 42], which is developed from Ripley’s *K*-function. Ripley’s *K*-function is based on two-dimensional coordinate points of individuals and was used to analyze population distribution pattern and spatial association at different scales [41]. It is the most common method of population spatial distribution pattern analysis, and is estimated from the function K(r):

$$K(r)=\frac{A}{n^{2}}\sum_{i=1}^{n}\sum_{j=1}^{n}\frac{1}{W_{ij}}Ir(u_{ij}),(i\neq j)$$ (1)

Where *A* is the sample plot area, *n* is the total number of plant individuals, *u*_ij_ is the distance between point *i* and point *j*, when *u*_*ij*_≤*r*, *Ir(u*_*ij*_*)* = 1, when u_ij_≥*r*, *Ir(u*_*ij*_*)* = 0; *W*_ij_ is the ratio of the arc length of the circle with point *i* as the center and *u*_*ij*_ as the radius in area A to the whole circumference, which can correct the error caused by boundary effect.

The expression of L(r) function is:

$$L(r)=\sqrt{\frac{K(r)}{\pi }}-r$$ (2)

when L(r) = 0, it indicates that the population is randomly distributed, when L(r) > 0, the distribution is aggregated, and when L(r) < 0, it is uniformly distributed.

Bivariate K_12_(r) function and modified L_12_(r) were used to analyze the spatial correlation of different age groups and different populations. The calculation formulas are as follows:

$$K_{12}(r)=(\frac{A}{n_{1}n_{2}})\sum_{i=1}^{n}\sum_{j=1}^{n}\frac{1}{W_{ij}}Ir(u_{ij}),$$ (3)

$$L_{12}(r)=\sqrt{\frac{K_{12}(r)}{\pi }}-r$$ (4)

where *n*_1_ and *n*_2_ are the total number of individuals in species 1 (or seedlings) and species 2 (or adults), respectively, other symbolic meanings are the same as in Formulas (3) and (4). When L_12_(r) = 0, the two populations had no correlation at r scale, when L_12_(r) > 0, the two populations were positively correlated at the *r* scale, when L_12_(r) < 0, the two populations were negatively correlated at *r* scale.

The Monte−Carlo fitting test was used to randomly simulate 1000 iterations to calculate the upper and lower envelopes, with a confidence level of 99%. For each *r* value, the values of L(r) and L_12_(r) were calculated and the process repeated until the predetermined number of iterations was reached. The L(r) value falls above the envelope line as an aggregated distribution, is randomly distributed within the envelope line, and is uniformly distributed below the envelope line. For the spatial association of the two populations, the value of L_12_(r) indicates a positive association above the envelope, no association between the envelopes, and a negative association below the envelope. All the special distribution calculations were performed using ADE-4 package (http://pbil.univ-lyon1.fr/ADE-4/) [43].

## Results

### Community characteristics and wind erosion

**Fig 2:**
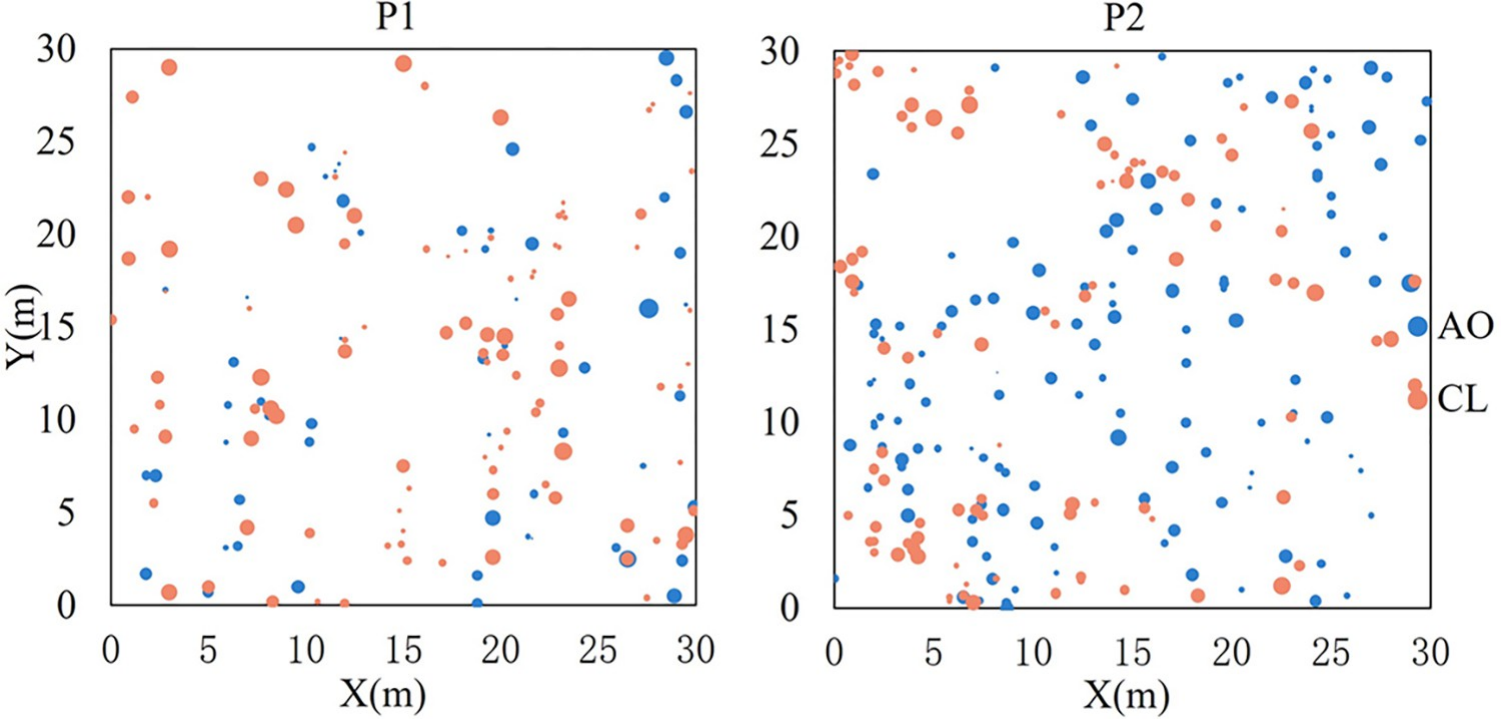
Mapped point pattern of *C*. *lignosum* var. *leave* and *A*. *ordosica* in sampling sites. AO: *A*. *ordosica*, CL: *C*. *lignosum* var. *leave*. Symbol sizes represent the crown of individuals.

The individual number of *A*. *ordosica* and *C*. *lignosum* var. *leave* was significantly different in the two restoration patterns, with 157 individuals mapped for the P1 and 231 for the P2 (Fig 2), and coverage of 17.04% and 22.62%, respectively, accounting for 70.20% and 86.45% of the total coverage of the community. In P1, there were 56 and 101 individuals of *A*. *ordosica* and *C*. *lignosum* var. *leave*, respectively, while in P2, their numbers 134 and 97. This suggests that P1 is more conducive to colonization by *C*. *lignosum* var. *leave*, and P2 is more suitable for colonization by *A*. *ordosica*. There was no significant difference in their growth between the two patterns. In P1 and P2, the seedlings of colonized shrubs accounted for 40.76% and 26.84% of the total individuals. The proportion of seedlings indicates that the population is in the growth stage. In addition, a small number of annual herbs such as *A*. *squarrosum* and *C*. *hyssopifolium* appeared in the two plots.

**Fig 3:**
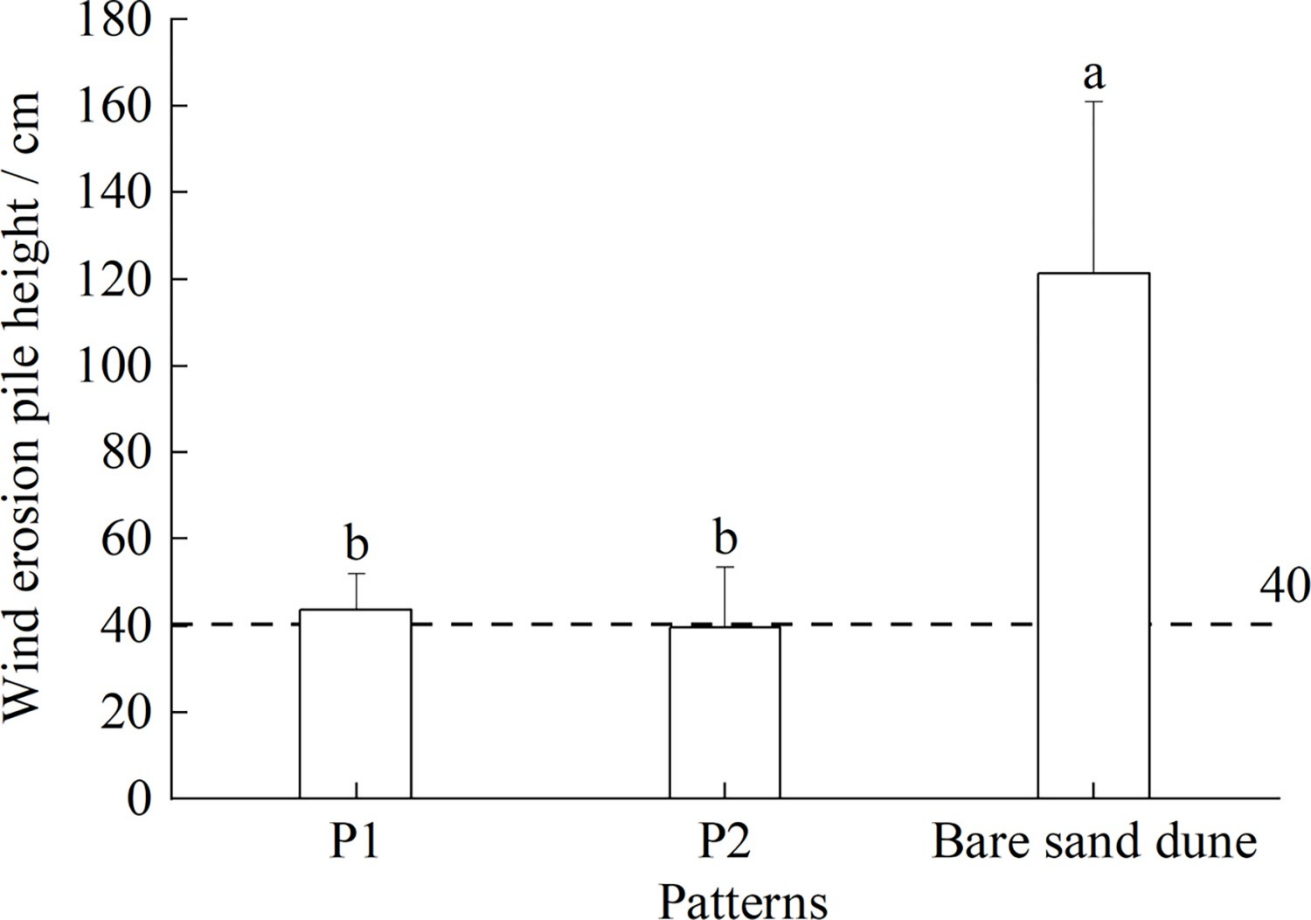
Wind erosion of sample plot. Dash line indicates the initial height of the wind erosion piles.

The results of wind erosion measurement showed that the average wind erosion amount of P1, P2 and mobile sand were -3.67 cm, 0.33 cm and -81.35 cm. There was no significant difference in wind erosion amount between the two vegetation restoration modes, but it was significantly lower than that of the mobile sand (*P* < 0.05) (Fig 3). The survey also found that although there were some residual Salix sand barriers and litters on the ground in P1, there was still obvious wind erosion, while P2 generally formed obvious sand ridges at the downwind of sand barriers due to the better wind-proof effect of *C*. *mongolica* living sand barriers, but the wind erosion in the plot was uneven and had obvious heterogeneity. The mobile sand was set in the downwind direction above the two restoration modes. Because the artificial vegetation changed the direction and size of the wind, the top of the dune at the downwind mouth was flattened, and the wind erosion was aggravated. The amount of wind erosion was significantly higher than that of the two vegetation restoration modes.

### Spatial pattern analysis of colonized shrubs

**Fig 4:**
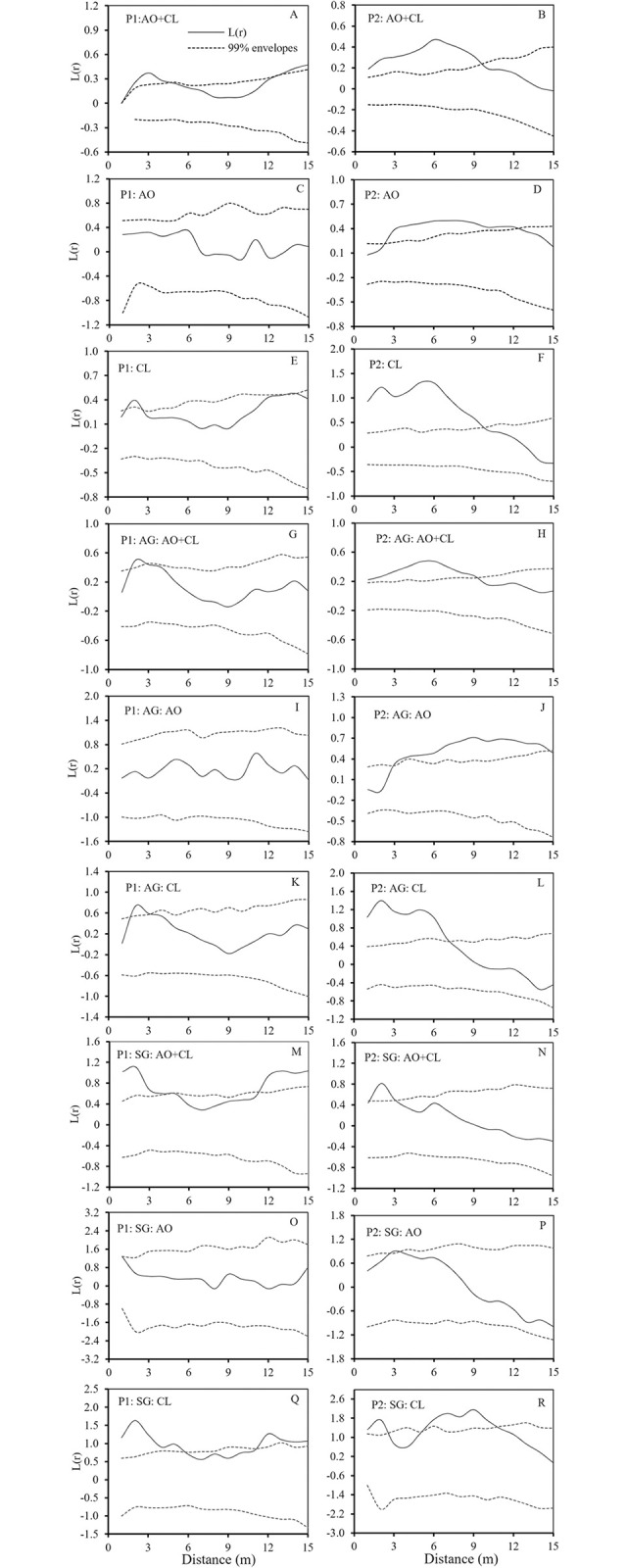
Spatial distribution patterns of *A*. *ordosica* and *C*. *lignosum* var. *leave* in two restoration patterns. AG: adult group, SG: seedling group, AO: *A*. *ordosica*, CL: *C*. *lignosum* var. *leave*. The solid line indicates the L(r), and the dashed lines represent the confidence intervals.

In P1, all *A*. *ordosica* and *C*. *lignosum* var. *leave* individuals were distributed randomly at a scale of 4–12 m and aggregated at smaller and larger scales. (Fig 4A). Seedlings were randomly distributed at 5–11 m, and aggregated at other scales, while adults were randomly distributed at the study scale (Fig 4M and 4G). In P2, all individuals were aggregated below 10 m and were randomly distributed at a larger scale (Fig 4B). The seedlings were below 3m, and the adults were below 9m, showing aggregated distribution, and the larger scale was randomly distributed (Fig 4N and 4H). In P1, all individuals, adult and seedling, of *A*. *ordosica* were distributed randomly at all scales (Fig 4C, 4I and 4O), whereas in P2, seedlings were distributed randomly at all scales (Fig 4P), but adult individuals were only distributed randomly below 3 m, and aggregated on a larger scale (Fig 4J). All individuals of *A*. *ordosica* were aggregated at 2–13 m, and randomly distributed at other scales (Fig 4D). *C*. *lignosum* var. *leave* was randomly distributed at all scales in P1 (Fig 4E), but all individuals, adults and seedlings, were aggregated at scales below 7–10 m and randomly distributed at larger scales in P2 (Fig 4F, 4L and 4R). In P1, adult individuals were randomly distributed at all scales (Fig 4K), seedlings were randomly distributed at 5–10 m, and aggregated at smaller and larger scales (Fig 4Q). In P2, adult individuals were aggregated below 9 m while seedlings were aggregated below 3 m and randomly distributed at a larger scale (Fig 4F and 4R).

### Intra- and interspecific spatial associations

**Fig 5:**
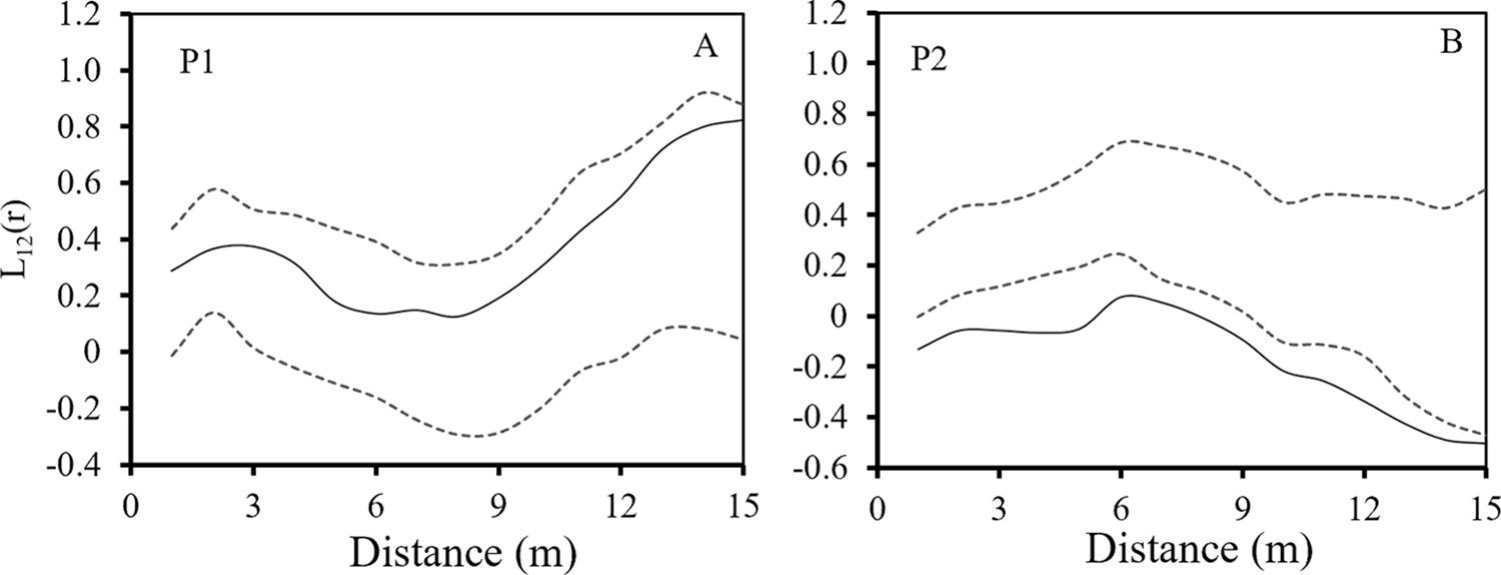
Spatial association of *A*. *ordosica* and *C*. *lignosum* var. *leave* in two restoration patterns.

**Fig 6:**
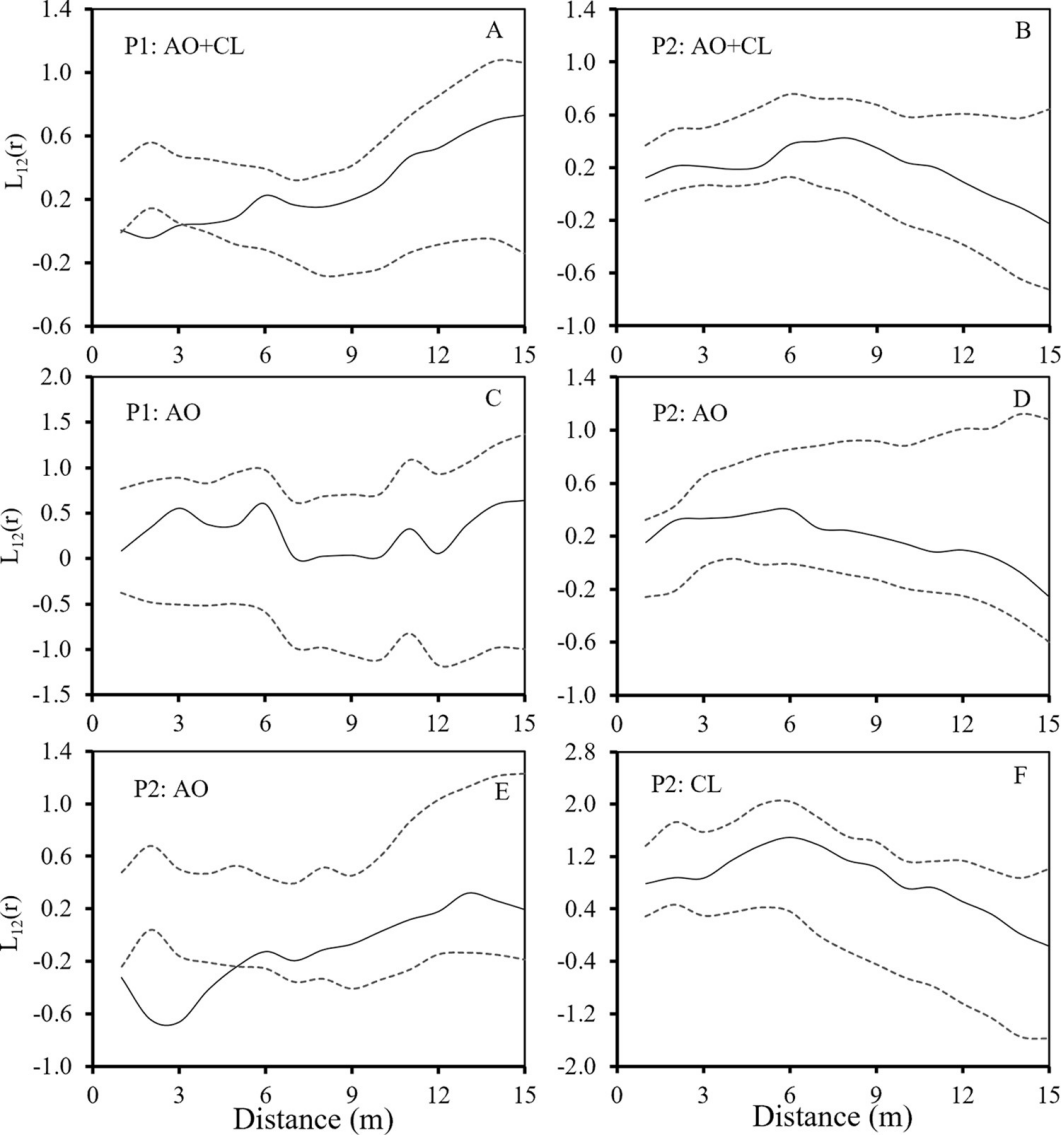
Association between seedlings and adults of *A*. *ordosica* and *C*. *lignosum* var. *leave* in two restoration patterns.

The association analysis included the spatial association of adult and seedling *A*. *ordosica* and *C*. *lignosum* var. *leave*. Under the two restoration patterns, there were differences in the spatial associations of *A*. *ordosica* and *C*. *lignosum* var. *leave*. P1 had no spatial association (Fig 5A), but P2 had negative spatial association (Fig 5B). In the two restoration patterns, the spatial association between all seedlings and adults of *A*. *ordosica* and *C*. *lignosum* var. *leave* was different (Fig 6A and 6B). In the two restoration patterns, there was no spatial correlation between the adults and seedlings of *A*. *ordosica* (Fig 6C and 6D). The analysis of spatial association between adults and seedlings of *C*. *lignosum* var. *leave* showed negative association within 5 m and no association above 5 m in P1 (Fig 6E). In P2, there was no spatial association between adults and seedlings (Fig 6F).

## Discussion

### Effects of artificial measures on natural restoration of native shrubs

Our study demonstrated that artificial measures such as low-density tree afforestation and the construction of living shrub sand barriers could promote the colonization by native shrubs and quickly fix mobile dunes during the early stages of vegetation restoration. Sandy land in semi-arid areas degraded below a certain threshold could not be naturally restored, and artificial measures were required to re-establish vegetation [44]. Studies had shown that the construction of straw checkerboards and shrub seedlings plantations could significantly improve topsoil development on the dune surface, as well as accelerate the plant diversity, vegetation cover, and plant density [11]. A sand dune was a dynamic environment, determined by wind erosion, sand accumulation, and dune encroachment, which directly impacted the spatial distribution and growth of plants [45, 46]. We found that planting native trees (*S*. *matsudana*) at low densities (about 50% lower than conventional afforestation) and low coverage of living sand barriers (*C*. *mongolica*) could provide a nurse effect to facilitate the colonization by *A*. *ordosica* and *C*. *lignosum* var. *leave*. After four years, the cover of the two native shrubs in the two plots had reached 17.04% and 22.62%, respectively. *A*. *ordosica* and *C*. *lignosum* var. *leave* remained the dominant species in Mu Us sandy land [35], and their colonization was conducive to the formation of more stable near-natural vegetation.

The interactions between plants and their environment determined the composition and structure of plant communities and affected their dynamics [18, 47]. Artificial plantings provided a living environment for the invasion of native plants in the early stages of vegetation restoration by changing the microenvironment through wind speed reduction, sand flow reduction, seed capture, and seedling protection [30, 48]. The number and distribution patterns of *A*. *ordosica* and *C*. *lignosum* var. *leave* in different restoration projects were different in this study (Table 1 and Fig 2), a finding consistent with previous research [49]. In the fourth year of vegetation restoration, it was observed that the living shrub sand barrier of *C*. *mongolica* was more conducive to the colonization and settlement of *A*. *ordosica* and *C*. *lignosum* var. *leave* than the *S*. *matsudana* plantation pattern. This was due to the dwarf shrub *C*. *mongolica* being closer to the ground and forming a dense barrier, effectively reducing wind speed and surface sand flow; the microenvironment created between rows was more favorable for seed capture and germination. The fallen branches and leaves of artificially planted trees formed a natural barrier under the trees, which helped seed capture and seedling recruitment, allowing native shrubs to grow near the trees (Fig 1). Regional vegetation without artificial measures did not show signs of recovery (Fig 1). This suggested that viable artificial vegetation reconstruction was crucial to the restoration of a degraded sandy ecosystem.

### Spatial patterns of native shrubs in two restoration patterns

Population structure and spatial pattern were the results of the interaction of biological characteristics, environmental factors, and interspecific competition, and they reflected population dynamics and community succession [47, 50]. Analysis of the spatial pattern and population structure at different stages of population growth could provide basic population information, such as recruitment, individual mortality, and intraspecies competition [33, 35].

*A*. *ordosica*, a native semi-shrub species, was the main dominant species that was broadly distributed in the Mu Us sandy land [10]. It adapted to the moving sand dune environment by changing the biomass allocation under burial and denudation stress [51]. This study showed that the adults and seedlings of *A*. *ordosica* were randomly distributed at all scales in PI, which may be primarily related to its reproductive mode. The seeds of *A*. *ordosica* were small and light, being suitable for wind dispersal, which could be over a significant distance. Seeds were captured by artificially planted vegetation, where they germinated and survived in the plot, a process that occured randomly. However, in P2, adult individuals were randomly distributed at small scales but clustered at larger scales (Fig 4J). These results were different from the previous research [10, 35]. Liu and Zhang studied the population pattern and structure of *A*. *ordosica* in enclosures and found that seedlings and adult populations showed a small-scale aggregation distribution with a random distribution at a large scale [35]. It was generally understood that small scale aggregation distribution was beneficial for seedlings as it assisted them to resist adverse natural conditions [52]. In this study, the sand barrier changed wind speed and sand burial, thereby influencing the distribution pattern of *A*. *ordosica*. The main reason for this difference might be that the artificial restoration model of *C*. *mongolica* sand barrier had an impact on the spatial distribution pattern of colonial native semi-shrubs. In addition, there were relatively more *A*. *ordosica* settled in the early stage of restoration, which was aggregated in a limited space, and the settlement growth time was shorter. *A*. *ordosica* had not yet entered the self-thinning stage of aging and death. Therefore, the spatial distribution pattern of the population was determined by many factors, such as human disturbance, intraspecific and interspecific competition, environmental factors and plant biological characteristics.

*C*. *lignosum* var. *leave* was a rhizomatous clonal semi-shrub widely dispersed by aerial seeding in Mu Us sandy land in northern China since the 1950s. The high clonal propagation and population growth rate of *C*. *lignosum* var. *leave* enabled it to quickly colonize in mobile dunes and to realize rapid spatial expansion [53]. The spatial distributions of the two patterns were different in this study. Adults were randomly distributed in P1, whereas in P2 they were aggregated within 7 m and randomly distributed as the scale increased (Fig 4K and 4L). The nurse effect of planted trees created an appropriate microenvironment for the seed germination and settlement of *C*. *lignosum* var. *leave*. As a result, the distribution of adult *C*. *lignosum* var. *leave* was more similar to that in the natural environment and was random. In P2, due to the height limitation of the shrub sand barrier, the nurse effect range was limited, meaning *C*. *lignosum* var. *leave* aggregation occurred only within a certain range (Fig 4R). This was also related to the growth status of *C*. *mongolica* in the plot, including height differences and sand barrier death fragments. Studies showed that populations of *C*. *lignosum* var. *leave*, once established, were able to realize rapid population expansion through high clonal propagation [53]. The first settled individuals produced more seedlings through asexual reproduction in its surroundings, resulting in seedlings aggregated on a small scale.

In comparison to the mechanical sand barrier, the plant sand barrier used living plants as raw materials, which significantly shortened the vegetation restoration cycle, and had the advantages of low cost, rapid effect, high stability, and strong ecological function in the practice of desertification control. In this study, a living sand barrier with a large row spacing (5 m) was used, which not only reduced costs but also provided enough space for the colonization by native shrubs, thereby increasing species diversity. These were also the most significant differences compared to conventional dense grid sand barriers. The study also found that living sand barriers affected the spatial distribution pattern of plants by controlling the direction and speed of wind, resulting in changes in the erosion and deposition patterns. Therefore, afforestation species and configuration methods had an important impact on the spatial patterns of colonized plants. Once the native shrubs were planted, they gradually became nurse plants and promoted rapid vegetation restoration.

The differences in the spatial distribution patterns of colonized native semi-shrubs in these two restoration patterns could be related to the life form of planted plants, configuration methods and biological characteristics of colonized plants. *S*. *matsudana* and *C*. *mongolica* were native tree species in Mu Us sandy land, which had good windbreak and sand fixation function and adaptability. The results of this study showed that the colonial native semi-shrubs tended to be more randomly distributed at P1 and more clustered at P2. This might be related to the fact that *S*. *matsudana*, as a tree, had thin and high branches, and its wind erosion resistance was lower than that of low semi-shrub *C*. *mongolica*. Moreover, *C*. *mongolica* was planted in sand barrier mode, and its wind erosion resistance function was strong (Fig 3). In addition, there were also differences in the spatial distribution of wind erosion and sand burial in the two plots. The wind erosion under the *S*. *matsudana* forest was more uniform and dominated by wind erosion, while the *C*. *mongolica* was lower and formed a sand ridge in the downwind near the sand barrier. The uneven distribution of erosion and sand burial increased heterogeneity, which was also conducive to the settlement of native colonial plants and also affected its distribution pattern. In summary, the differences in morphological characteristics and planting patterns of *S*. *matsudana* and *C*. *mongolica* leaded to wind erosion and sand burial patterns in the plots, thus affecting the spatial distribution pattern and quantity of colonial native semi-shrubs.

### Intra- and interspecific spatial association of colonial native shrubs

Plant-plant interactions were a major driving force in plant community succession or vegetation restoration [54]. Positive (promotion) and negative (competition) effects existed and occured simultaneously between plants in the community, and the environmental conditions determined which effect was more important [26]. The positive effect of nurse plants increased under stressful abiotic conditions, which were evidently greater in higher mountains, particularly during the earlier stages of degraded ecosystem restoration. As the main type of facilitation, nurse effect was used widely in ecological restoration of degraded habitats [55], and it can effectively shorten the recovery time. Nurse plants primarily improved the surrounding microenvironment, enabling adjacent species to settle in their original environment. This effect was particularly important in areas with harsh environmental conditions, such as arid and semi-arid areas, alpine areas, and salt marshes [25, 56]. Plants that were the first established in an area will impact the recruitment, growth, and reproductive success of species that arrive later [57]. This study also supported the stress gradient hypothesis. The vegetation established by different artificial measures in the harsh semi-arid sandy environment played the role of nurse plants and promoted the colonization by native shrubs *A*. *ordosica* and *C*. *lignosum* var. *leave*.

The stress gradients and biological characteristics of nurse and target plants determined the results of plant interactions, which in turn affected the success or failure of vegetation restoration [58]. The target plants in this study were native shrubs and herbs, and they were selected because the establishment of near-natural communities was the key to vegetation restoration success. In the early stage of sand dune vegetation restoration, both restoration methods promoted the colonization by native shrubs *A*. *ordosica* and *C*. *lignosum* var. *leave*. The spatial distribution pattern and spatial association of colonial shrubs could explain the facilitation mechanism of nurse plants [59]. Nurse plants could make the probability of target plants appearing under their canopy higher than those on open land [60], and their survival rates in different directions were also significantly different [61], thus affecting the spatial distribution pattern of target plants. In this study, there was no association between the populations of *C*. *lignosum* var. *leave* and *A*. *ordosica* in P1. Tree stems were thin and tall, forming different synusia structures in *C*. *lignosum* var. *leave* and *A*. *ordosica*, and had no direct competition with shrubs. The space and resources in the sample plot were sufficient and *A*. *ordosica* and *C*. *lignosum* var. *leave* could grow in their own spaces, with no evident interspecific competition. *A*. *ordosica* and *C*. *lignosum* var. *leave* had a negative association in P2 (Fig 5B), because the spacing of *C*. *mongolica* sand barrier planting was small, and the age and individual size was similar to the target plant [62], indicating that the colonial shrubs were excluded from the inter row distribution. The number of individuals of *A*. *ordosica* and *C*. *lignosum* var. *leave* was greater than in P1, and their spatial distribution pattern demonstrated aggregation. With community succession, the competition for space and resources between the two species increases. However, low-density (large row spacing) planting methods not only minimized the direct competition between nurse plants and target plants in the early stages of sand vegetation restoration, but also provided suitable seed germination, seedling recruitment, and growth conditions for the colonization by native plants in our study. Low-density planting methods not only effectively resolved competition between artificially planted vegetation and native plants in the early stages of vegetation restoration, but also improved the effectiveness of vegetation restoration. Other studies in the study area concluded that dune stabilization can change the spatial pattern of shrub populations by weakening the spatial association between native shrub individuals [63].

The spatial association analysis between individuals at different growth stages could help us to explain the mechanism of spatial pattern formation in the early stages of vegetation restoration [64]. *C*. *lignosum* var. *leave* and *A*. *ordosica* were dominant plants in different succession stages in Mu Us sandy land. *C*. *lignosum* var. *leave* was a typical guerrilla clonal plant, and clonal reproduction plays an important role in its life history, while *A*. *ordosica* mainly relied on seed reproduction to maintain population and population diffusion. The results of this study showed that different ecological restoration methods strongly affected the spatial association of colonial native shrubs, and that there was no spatial association between adults and seedlings of *A*. *ordosica* (Fig 6C and 6D). In P1, *C*. *lignosum* var. *leave* adults and seedlings showed a negative spatial correlation on a smaller scale, which was contrary to the results of natural population studies [35]. These results were due to the nurse effect observed in different restoration methods, and the ecological strategy of colonizing shrubs may jointly affect the spatial pattern of the population. First, there were many natural *A*. *ordosica* distributed around the plot. The seeds of *A*. *ordosica* were small and light, which were easy to reach the plot in the wind. Secondly, the seeds had mucilage, and the germination water demand was small [65, 66]. These breeding strategies adapted to the arid sandy environment maked it quickly settle in the plot, and the establishment process tended to be random. The reproductive strategy of *C*. *lignosum* var. *leave* was different from that of *A*. *ordosica*. Even if the number of colonies was small, it relied on its strong rhizome cloning ability to quickly occupy limited resources and expanded the population size. In the two restoration modes, the spatial correlation between adult and seedling of *C*. *lignosum* var. *leave* was negatively correlated at a small scale (5m). This may be a strategy that when the diffusion space was large enough in the early stage of vegetation restoration, the clonal foraging behavior of *C*. *lignosum* var. *leave* made the daughter plant colonize as far as possible from the genet to avoid premature competition. In this study, a small number of *C*. *lignosum* var. *leave* seeds may first settle in the plot and start clonal reproduction 3–4 years later. Or at the same time, the rhizomes of *C*. *lignosum* var. *leave* around the plot entered the plot for clonal reproduction [37, 63]. However, the landscape heterogeneity and the species pool in the restoration area also affected the restored species. Sufficient seed sources around the restoration area were a prerequisite for the colonization by native plants.

In this study, both vegetation restoration models promoted the colonization of native shrubs *A*. *ordosica* and *C*. *lignosum* var. *leave*, and had different effects on the spatial distribution pattern of the two colonial native semi-shrubs. However, the process of vegetation restoration was a dynamic process. With the succession of the community, the intra- and interspecific relationships would continue to change. It was not enough to study the population pattern at a certain time point. Therefore, we recommended that future studies should be carried out from more time series to grasp the whole process of vegetation restoration in order to better guide production activities.

## Conclusion

This study provided novel insights into the ecological restoration of degraded sandy ecosystems by analyzing the spatial patterns of early colonized semi-shrubs, *A*. *ordosica* and *C*. *lignosum* var. *leave*, in two restoration patterns. In the early stages of restoration, the nurse effect of artificial plantings in the two restoration patterns of low-density artificial afforestation of *S*. *matsudana* and the living sand barrier of *C*. *mongolica* promoted the colonization by local semi-shrubs *A*. *ordosica* and *C*. *lignosum* var. *leave*. Two restoration patterns have important effects on the number and spatial distribution pattern of colonizing native shrubs. In *S*. *matsudana* plantation pattern, the spatial distribution pattern of colonial shrubs tended to be random, and there was no spatial association between species. In the *C*. *mongolica* living sand barrier pattern, the colonial shrubs aggregation distribution was more dominant, and with the increase of scale, the aggregation distribution changed to random distribution, while the interspecific association was negatively correlated. In the ecological restoration of degraded sandy land, the life form and configuration of plant species and the biological characteristics of colonial plants jointly affected the community structure and succession process. This study clearly demonstrated that low-cost restoration measures could achieve better restoration results in ecological restoration of sandy land in semi-arid areas. As it was necessary to evaluate the promotion effect of different artificial measures on dominant native plants to improve the effectiveness of vegetation restoration, our results provided a theoretical basis for ecological restoration of degraded sandy land.
